# Macrolide and lincosamide resistance of *Streptococcus agalactiae* in pregnant women in Poland

**DOI:** 10.1038/s41598-024-54521-y

**Published:** 2024-02-16

**Authors:** Dorota Kamińska, Magdalena Ratajczak, Dorota M. Nowak-Malczewska, Justyna A. Karolak, Marek Kwaśniewski, Anna Szumala-Kakol, Jolanta Dlugaszewska, Marzena Gajecka

**Affiliations:** 1 Chair and Department of Genetics and Pharmaceutical Microbiology, Collegium Pharmaceuticum, Poznan University of Medical Sciences, Rokietnicka 3, 60-806 Poznan, Poland; 2 Institute of Human Genetics, Polish Academy of Sciences, Strzeszynska 32, 60-479 Poznan, Poland; 3 Unit of Microbiology, Gynecological and Obstetric Clinical Hospital, Poznan University of Medical Sciences, Polna 33, 60-535 Poznan, Poland

**Keywords:** Group B Streptococcus, Macrolide resistance, Lincosamide resistance, Streptogramin resistance, Antimicrobial susceptibility patterns, Serotyping, Virulence factors, Clinical microbiology, Bacterial infection

## Abstract

Knowing about the antibiotic resistance, serotypes, and virulence-associated genes of Group B Streptococcus for epidemiological and vaccine development is very important. We have determined antimicrobial susceptibility patterns, serotype, and virulence profiles. The antibiotic susceptibility was assessed for a total of 421 *Streptococcus agalactiae* strains, isolated from pregnant women and neonates. Then, 89 erythromycin and/or clindamycin-resistant strains (82 isolates obtained from pregnant women and seven isolates derived from neonates) were assessed in detail. PCR techniques were used to identify the studied strains, perform serotyping, and assess genes encoding selected virulence factors. Phenotypic and genotypic methods determined the mechanisms of resistance. All tested strains were sensitive to penicillin and levofloxacin. The constitutive MLS_B_ mechanism (78.2%), inducible MLS_B_ mechanism (14.9%), and M phenotype (6.9%) were identified in the macrolide-resistant strains. It was found that macrolide resistance is strongly associated with the presence of the *ermB* gene and serotype V. *FbsA, fbsB, fbsC, scpB*, and *lmb* formed the most recurring pattern of genes among the nine surface proteins whose genes were analysed. A minority (7.9%) of the GBS isolates exhibited resistance to lincosamides and macrolides, or either, including those that comprised the hypervirulent clone ST-17. The representative antibiotic resistance pattern consisted of erythromycin, clindamycin, and tetracycline resistance (71.9%). An increase in the fraction of strains resistant to macrolides and lincosamides indicates the need for monitoring both the susceptibility of these strains and the presence of the ST-17 clone.

## Introduction

*Streptococcus agalactiae* (Group B Streptococcus, GBS) inhabits the gastrointestinal and genital tracts as a component of the average human microbiota. However, this species is also associated with various infections affecting newborns, pregnant or postpartum women, and people over the age of 60. The most common diseases in newborns and neonates caused by GBS are pneumonia, sepsis, and meningitis^1^. Diagnosing skin and soft tissue infections, bacteremia, pneumonia, and osteoarthritis in adults is frequent. GBS is also a known etiologic agent of urinary tract infections, including pyelonephritis and prostatitis^2–4^.

GBS strains are universally sensitive to penicillin, with rare isolates with minimum inhibitory concentrations (MIC) at or above the susceptible breakpoint of 0.12 µg/mL^5–7^. Clindamycin is the recommended antibiotic alternative for patients with a history of allergy to β-lactams. However, there has been a recent increase in the identification of clinical GBS strains resistant to these antibiotics^8^. In GBS, resistance to macrolides arises mainly from an active drug efflux controlled by the *mefA* gene or modification of the drug target on the rRNA through methylases encoded by *erm* genes. Resistance can be expressed as a cross-resistance to macrolides, lincosamides, and streptogramin B (inducible-iMLS_B_ and constitutive-cMLS_B_) or a resistance to 14- and 15-membered ring macrolides only, while lincosamides, streptogramins, and 16-membered ring macrolides remain active (phenotype M). In addition, the clindamycin-resistant erythromycin-susceptible phenotype (L phenotype) has been reported^9–11^. According to the latest Centers for Disease Control and Prevention (CDC)’ report, clindamycin-resistant GBS belongs to the category of “Concerning Threats”, based on the hazard to human health^12^. In pregnant women who are allergic to penicillin, it is crucial to consider the sensitivity of GBS to erythromycin and clindamycin. In such cases the perinatal prophylaxis, which includes ampicillin, penicillin, or cefazolin, cannot be administered.

GBS strains exhibit some virulence factors that are important during adhesion, invasion, and avoidance responses from the host's immune system. Surface proteins, including laminin-binding protein (Lmb), serine repeat proteins (Srr), fibrinogen-binding proteins (FbsA, FbsB, and FbsC), Rib, and alpha C, allow bacteria to adhere to host cells, which is crucial for further colonization or invasion of the host organism^13,14^. Capsular polysaccharides are one of the most essential factors of GBS virulence. Ten serotypes (Ia, Ib, II-IX) have been distinguished based on differences in the structure of capsular polysaccharides. The occurrence of particular serotypes varies, depending on the geographic region of the world and ethnicity, and also varies over time for a given region^4,15–18^. Previously, 98% of worldwide identified GBS isolates were found as serotypes Ia, Ib, II, III, IV, and V^15^. In the US, these six predominant serotypes were accountable for over 99% of early-onset and late-onset GBS-related diseases^8^. The systematic review demonstrated that serotype III, associated with invasive disease, accounts for 25% of observations but is less common in some South America and Asia countries, where serotypes VI-IX are more frequently identified^15^. In particular, serotype III isolates belonging to the sequence type (ST) 17 lineage (ST-17) have been defined as hypervirulent. This strain possesses numerous virulence factors, including a surface-anchored protein called hypervirulent GBS adhesin (HvgA). The *Gbs2018/hvgA* gene encoding this adhesin is being efficiently used to identify the ST-17 strains^19,20^. Estimating the prevalence of particular serotypes and surface proteins in different geographic regions is crucial as they will become targets for future vaccination, particularly for vaccines under development^4,16^.

The aim of this study was to determine antimicrobial susceptibility patterns, serotype, and virulence gene profiles of GBS isolates with macrolide and/or lincosamide resistance to highlight the increasing resistance to these groups of antibiotics in pregnant women.

## Results

### cMLS_B_ found as a dominant phenotype

Of the evaluated 421 strains, 89 (21.1%) isolates were resistant to erythromycin and/or clindamycin and were subjected to further characterization. Among them, 82 were isolated from vaginal or rectal swabs from pregnant women and seven were derived from newborns [blood (n = 4), urine (n = 2), and pharyngeal swabs (n = 1)]. All the erythromycin- and/or clindamycin-resistant strains were sensitive to penicillin and levofloxacin. Resistance to tigecycline was identified in one strain obtained from pregnant women. This strain was also resistant to tetracycline, macrolide, lincosamide, and streptogramin B (cMLS_B_ phenotype). All but three isolates from pregnant women showed resistance to tetracycline (96.6%). 71.9% (n = 64/89) of strains were simultaneously resistant to erythromycin, clindamycin, and tetracycline. Antibiotic resistance profiles are presented in Table 1.

**Table 1:** Virulence gene profile and resistance patterns among strains of GBS resistant to erythromycin and/or clindamycin.

Genotype	Number (%) of strains
Virulence gene profile	
*fbsA-fbsB-fbsC-lmb-scpB-alp2/3*	34 (38.2)
*fbsA-fbsB-fbsC-lmb-scpB-rib*	15 (16.9)
*fbsA-fbsB-fbsC-lmb-scpB-rib-hvgA*	7 (7.9)
*fbsA-fbsB-fbsC-lmb-scpB-bca*	10 (11.2)
*fbsA-fbsB-fbsC-lmb-scpB-epsilon*	7 (7.9)
*fbsA-fbsB-fbsC-scpB-alp2/3*	8 (9.0)
*fbsA-fbsB-fbsC-scpB-rib*	2 (2.2)
*fbsA-fbsB-fbsC-scpB-epsilon*	1 (1.1)
*fbsA-fbsB-fbsC-lmb-rib*	1 (1.1)
*fbsA-fbsB-fbsC-rib*	1 (1.1)
*fbsA-fbsB-fbsC-epsilon*	2 (2.2)
*lmb-scpB-rib*	1 (1.1)
Resistance patterns	
E-C-Te-Tig	1 (1.1)
E-C-Te	64 (71.9)
E-C	3 (3.4)
E-Te	19 (21.3)
C-Te	2 (2.2)

*E* erythromycin, *C* clindamycin, *Te* tetracycline, *Tig* tigecycline.

**Figure 1:**
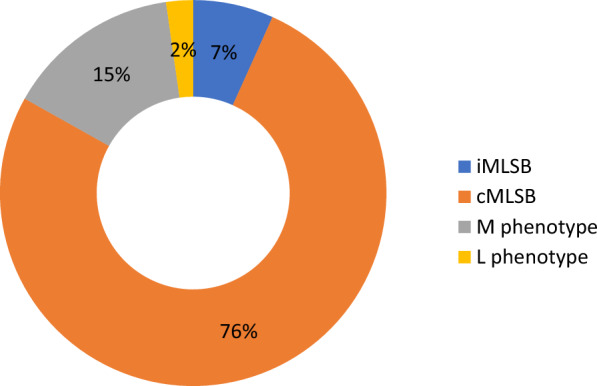
Occurrence of resistance phenotypes among 89 strains of GBS resistant to erythromycin and/or clindamycin.

Comparing the occurrence of macrolide, lincosamide, and streptogramin B resistance mechanisms among the tested strains, the iMLS_B_ was identified in 6.74% of strains (n = 6/89; all from pregnant women), cMLS_B_ was found in 76.4% of strains (n = 68/89; 64 from pregnant women, four from newborns), the M phenotype was found in 14.6% of strains (n = 13/89; 10 from pregnant women, three from newborns), whereas the L phenotype was identified in 2.25% of GBS strains (n = 2/89; all from pregnant women) (Fig. 1).

### The presence of the ermB gene is a major determinant of erythromycin resistance

In all strains belonging to serotype Ia, the M phenotype (efflux mechanism), determined by the presence of the *mefA* gene, was found. In contrast, all strains belonging to serotypes Ib and IV showed the cMLS_B_ phenotype, with resistance to clindamycin and erythromycin. The cMLS_B_ phenotype predominated also in strains belonging to serotypes III and V [71.4% (n = 10/14) and 90.4% (n = 47/52) of strains, respectively], followed by the iMLS_B_ [14.3% (n = 2/14) and 5.8% (n = 3/52)], and the M phenotype [14.3% (n = 2/14) and 1.9% (n = 1/52)]. The observed differences in prevalence of resistance mechanisms among different serotypes were statistically significant (p = 5.508e−16). Among 68 isolates with the cMLS_B_ phenotype, 94.1% of samples (n = 64/68) contained the *ermB* gene, 4.4% of samples (n = 3/68) had the *ermA* gene, while the remaining 1.5% of samples (n = 1/68) contained both the *ermB* and *mefA* genes. All isolates with the iMLS_B_ phenotype contained the *ermA* gene alone (n = 6/6), while isolates with the M phenotype contained the *mefA* gene (n = 13/13). In the two strains with the L resistance phenotype, the presence of the *lnuB* and *lsaE* genes was confirmed. These strains, one with serotype V and the other with serotype VI, were derived from pregnant women. The profiles of virulence genes, *fbsA-fbsB-fbsC-lmb-scpB-rib* and *fbsB-fbsC-lmb-scpB-bca*, were identified in isolates with serotypes V and VI, respectively.

The resistance genotypes and phenotypes identified among strains from pregnant women and newborns are presented in Table 2.

**Table 2:** Distribution of resistance phenotypes and genotypes among GBS strains with particular serotypes with erythromycin and clindamycin-resistances, or either, in pregnant women and newborns.

Serotype	Antibiotic resistance genes						Resistance phenotype			
	*ermB*	*ermA*	*mefA*	*tetM*	*lnuB*	*lsaE*	cMLS_B_	iMLS_B_	M	L
Pregnant women (n = 82)										
Ia (n = 8)	0	0	8 (100)*	2 (25.0)	0	0	0	0	8 (100)	0
Ib (n = 3)	3 (100)	0	0	0	0	0	3 (100)	0	0	0
II (n = 6)	5 (83.3)	1 (16.7)	0	2 (33.3)	0	0	5 (83.3)	1 (16.7)	0	0
III (n = 13)	9 (69.2)	3 (23.1)	2 (15.4)	3 (23.1)	0	0	10 (76.9)	2 (15.4)	1 (7.7)	0
IV (n = 2)	0	2 (100)	0	0	0	0	2 (100)	0	0	0
V (n = 49)	44 (89.8)	3 (6.1)	1 (2.0)	35 (71.4)	1 (2.0)	1 (2.0)	44 (89.8)	3 (6.1)	1 (2.0)	1 (2.0)
VI (n = 1)	0	0	0	0	1 (100)	1 (100)	0	0	0	1 (100)
Newborns (n = 7)										
Ia (n = 2)	0	0	2 (100)	0	0	0	0	0	2 (100)	0
II (n = 1)	1 (100)	0	0	0	0	0	1 (100)	0	0	0
III (n = 1)	0	0	1 (100)	1 (100)	0	0	0	0	1 (100)	0
V (n = 3)	3 (100)	0	0	2 (66.7)	0	0	3 (100)	0	0	0
Total (n = 89)	65 (73.0)	9 (10.1)	14 (15.7)	45 (50.6)	2 (2.2)	2 (2.2)	68 (76.4)	6 (6.7)	13 (14.6)	2 (2.2)

*The percentage values.

### Serotype V was recognized to be predominant

Analyzing the frequencies of particular serotypes among erythromycin- and/or clindamycin-resistant strains, it was found that more than half of these strains (58.4%, n = 52/89; 49 from pregnant women, three from newborns) belonged to serotype V, followed by serotypes III and Ia (15.7%, n = 14/89; 13 from pregnant women, one from newborn) and 11.2%,n = 10/89; eight from pregnant women, two from newborns, respectively) (Table 2).

#### The most common virulence gene pattern, including the hvgA gene

Distribution of each virulence-associated gene among the individual serotypes is shown in Table 3. Statistically significant differences were found only in the occurrence of the *rib* gene (p = 9.183e−08) among individual serotypes. The *fbsA*, *fbsB*, and *fbsC* genes were detected in 98.9% (n = 88/89; 81 from pregnant women, seven from newborns) of isolates. Most isolates carried the *lmb* and *scpB* genes (84.3% (n = 75/89; 71 from pregnant women, four from newborns) and 95.5% (n = 85/89; 79 from pregnant women, six from newborns), respectively), while *rib, bca, alp2/3*, and *epsilon* were present in 30.3% (n = 27/89; 25 from pregnant women, two from newborns), 11.2% (n = 10/89; all from pregnant women), 47.2% (n = 42/89; 40 from pregnant women, two from newborns), and 11.2% (n = 10/89; seven from pregnant women, three from newborns) of isolates, respectively. Virulence gene profiles are presented in Table 1. The most common gene pattern, including the *fbsA, fbsB, fbsC, scpB* and *lmb* genes, was recognized in 73 (82.0%) strains.

**Table 3:** Presence (frequency) of virulence related genes among erythromycin and clindamycin-resistant, or either, GBS strains in pregnant women and newborns.

Serotype	Virulence genes									
	*rib*	*bca*	*alp2/3*	*epsilon*	*lmb*	*scpB*	*fbsA*	*fbsB*	*fbsC*	*hvgA*
Pregnant women (n = 82)										
Ia (n = 8)	1 (12.5)*	2 (25.0)	0	5 (62.5)	6 (75.0)	8 (100)	8 (100)	8 (100)	8 (100)	0
Ib (n = 3)	0	2 (66.7)	1 (33.3)	0	3 (100)	3 (100)	3 (100)	3 (100)	3 (100)	0
II (n = 6)	4 (66.7)	2 (33.3)	0	0	6 (100)	6 (100)	6 (100)	6 (100)	6 (100)	0
III (n = 13)	11 (84.6)	2 (15.4)	0	0	12 (92.3)	12 (92.3)	13 (100)	13 (100)	13 (100)	7 (53.8)
IV (n = 2)	1 (50.0)	0	0	1 (50.0)	1 (50.0)	1 (50.0)	2 (100)	2 (100)	2 (100)	0
V (n = 49)	8 (16.3)	1 (2.0)	39 (79.6)	1 (2.0)	42 (85.7)	48 (97.9)	48 (97.9)	48 (97.9)	48 (97.9)	0
VI (n = 1)	0	1 (100)	0	0	1 (100)	1 (100)	1 (100)	1 (100)	1 (100)	0
Newborns (n = 7)										
Ia (n = 2)	0	0	0	2 (100)	1 (50)	1 (50)	2 (100)	2 (100)	2 (100)	0
II (n = 1)	0	0	0	1 (100)	1 (100)	1 (100)	1 (100)	1 (100)	1 (100)	0
III (n = 1)	0	0	1 (100)	0	1 (100)	1 (100)	1 (100)	1 (100)	1 (100)	0
V (n = 3)	2 (66.7)	0	1 (33.3)	0	1 (33.3)	3 (100)	3 (100)	3 (100)	3 (100)	0
Total (n = 89)	27 (30.3)	10 (11.2)	42 (47.2)	10 (11.2)	75 (84.3)	85 (95.5)	88 (98.9)	88 (98.9)	88 (98.9)	7 (7.9)

*The percentage values.

The occurrence of the ST-17 clone was found in 7.9% of the tested strains (n = 7/89). Five of the ST-17 clones had the cMLS_B_ phenotype, one had iMLS_B_ phenotype, and one had M phenotype. All of them were simultaneously resistant to tetracycline. Strains with the *hvgA* gene were isolated from pregnant women. No ST-17 clone was found in any of the seven neonatal strains.

## Discussion

Resistance of GBS strains to macrolides and lincosamides is discussed worldwide^6–8^. In this study, resistance to erythromycin and clindamycin was found in 20.7% (n = 87/421) and 16.6% (n = 70/421) strains, respectively. Previously, the fraction of strains resistant to erythromycin and clindamycin was recorded as variable and amounted to, respectively, 43.7% and 32.2% in Italy^21^, 30.0% and 28.0% in Switzerland^22^, and 14.5% and 14.0% in Sweden^23^. There is also a worrying trend toward increasing bacterial resistance to these groups of antibiotics seen in other populations^24–28^.

In line with currently reported data^6^, the cMLS_B_ resistance phenotype dominated (76.4%) among the evaluated erythromycin and clindamycin-resistant strains, or either. Other resistance phenotypes identified in our study, iMLS_B_ (6.7%) and M (14.6%), were also observed in 2.6% and 19.7% of isolates in an Italian population, respectively^6^. iMLS_B_ phenotype occurs when resistance to erythromycin induces resistance to clindamycin. This phenotype is detected in clinical laboratories, with a double-disc diffusion test (D-zone test) to prevent clinical treatment failure with clindamycin and the emergence of cMLS_B_ phenotype. The EUCAST guidelines recommend considering the iMLS_B_ phenotype of GBS to be clindamycin-resistant^29^. We detected the L phenotype in only two strains from pregnant women. These strains were susceptible to erythromycin but resistant to clindamycin. This resistance phenotype is rare in clinical GBS strains but has been previously reported in isolates from the United States^11^, Spain^30^, Argentina^31^, Korea^9^, and other countries^10,32,33^.

The study led by the CDC investigators in the US showed that all tested isolates (n = 1727) were susceptible to penicillin, ampicillin, and vancomycin, 44.8% had erythromycin resistance, and 20.8% had constitutive clindamycin resistance^8^. From 2006 to 2015, the proportion of erythromycin resistance increased significantly from 34.7 to 49.1%, whereas the constitutive clindamycin resistance increased from 14.7 to 26.0%^8^.

Most of the erythromycin and clindamycin-resistant strains, or either, evaluated in this study were found to be serotype V (58.4%). Previously, in the US, the proportion of isolates with erythromycin resistance was recorded as the highest for serotype II (erythromycin resistance 66.5%) and serotype V (erythromycin resistance 61.1%)^8^.

In this study, common genetic determinants of erythromycin resistance were the presence of the *ermB* (73.0%), *mefA* (16.1%), and *ermA* [subclass *erm*TR] (10.1%) genes. All strains presenting with the M phenotype exhibited the presence of the *mefA* gene, which supports the existing literature findings that indicate the *mefA* gene exclusively confers resistance to macrolides^32^. Moreover, 64 isolates showing the cMLB phenotype had only the *ermB* gene (94.1%). In comparison, three isolates with this phenotype contained only the *ermA* gene (4.4%). The *mefA* and *ermB* genes were simultaneously detected in one isolate with the cMLS_B_ phenotype (1.5%), suggesting the participation of two different genes in the appearance of macrolide and lincosamide resistance. Similar results have been found in the literature^10,32,34^. We observed a significant correlation between the presence of the *ermA* gene (100%) and the inducible resistance phenotype in our study. The available literature shows that the inductive phenotype is predominantly linked to a solitary *ermA* gene, although the existence of the *ermB* gene can also influence it^10,32^. Most of the clindamycin-resistant isolates showed cMLS_B_ phenotype determined by the *ermB* gene. However, two isolates had the L phenotype and were positive for the *lnuB* and *lsaE* genes, consistent with previous findings^9–11^.

Recent epidemiological reports indicate the spread of the multidrug-resistant (MDR) subclone ST-17 GBS characterized by the loss of Pl-1 [CC (clonal complex) 17/Pl-2b], simultaneously resistant to macrolides, lincosamides, and tetracycline and other antibiotics^20,35–39^. Teatero et al. identified 11 CC17 isolates in Canadian patients lacking Pl-1 and acquiring a mobile genetic element encoding resistance to tetracycline, macrolides, and other antibiotics at the same site^35^. Campisi et al. conducted a whole-genome sequencing analysis and antimicrobial susceptibility testing on 14 isolates with serotype III that are part of the hypervirulent CC17 in China. Thirteen of 14 isolates contained only the sequences encoding the Pl-2b, and in place of the Pl-1 operon, the multi-drug resistance gene clusters harbored in two new versions of integrative and conjugative elements were present^36^.

Similarly, European studies have reported the emergence of hypervirulent strains belonging to a sublineage characterised by the absence of Pl-1 (CC17/Pl-2b) and exhibiting multidrug resistance^20,37,38^. In Italy, GBS serotype III was predominant in early-onset (56%) and late-onset (95%) diseases. The resistance to clindamycine reached 29%, and most of the strains (76%) were serotype III-ST17 and contained the genetic markers of the MDR CC17 clone^20^. Studies conducted on 218 strains isolated from invasive infections in newborns in Portugal yielded comparable findings, in which 50% of the isolates were the CC17. The presence of the clone CC17/Pl-2b simultaneously resistant to macrolides, lincosamides, and tetracycline and exhibiting high-level resistance to streptomycin and kanamycin among the tested strains was confirmed^38^.

A small fraction of the GBS isolates resistant to macrolides and lincosamides, or either, consisted of the hypervirulent clone ST-17, (7.9%, n = 7/89). The presence of this clone was confirmed only among pregnant women (7/82). Thus, the increase in resistance to erythromycin and clindamycin in Poland is not due to the spread of the hypervirulent MDR ST-17 clone. However, the presence of strains belonging to this clone among pregnant women and the results cited above indicate the need to monitor the drug susceptibility of GBS strains and the frequency of the ST-17 clone.

As in other studies, also in this work, serotype V was dominant among the erythromycin and clindamycin-resistant, or either, strains^40–42^. Subsequently, the tested isolates were identified as serotypes III, Ia, II, Ib, IV, and VI. Two isolates with L phenotype belonged to serotypes V and VI. A similar virulence gene profile was found in these isolates, comprising the *fbsA, fbsB, fbsC, lmb*, and *scpB* genes. The isolates with the serotypes V and VI also contained the *rib* and *bca* genes, respectively. We detected the *fbsA, fbsB, fbsC*, and *scpB* genes in nearly all characterized isolates, and these genes exhibit an even distribution across various serotypes. These results are consistent with previous report, in which the most frequently detected genes were *scpB*, *fbsA*, *fbsB*, *cylB*, and *lmb*^43–46^. Also, studies utilizing the whole-genome sequencing showed that fibrinogen-binding protein, laminin-binding protein, and serine peptidase coding sequences were detected in ≥ 98% of the assessed samples. In comparison, the *rib* gene was identified in 27.8% of isolates^47^.

Healthcare providers utilise maternal screening and intrapartum antibiotic therapy as preventive measures against GBS infections in newborns. However, such early exposure to antibiotics alters the gut microbiota of infants and may have health consequences later in life.

As the frequency of strains resistant to macrolides, lincosamides, and streptogramin B is increasing worldwide, it is essential to characterize these isolates in detail. Epidemiological monitoring of GBS serotypes and their surface proteins is vital in a given geographic region. It plays a substantial role in vaccine design processes and future vaccination strategy implementation.

## Methods

### GBS strains collection and identification

A total of 421 GBS clinical strains isolated from vaginal or rectal swabs from pregnant women (n = 401) and newborns [(n = 20), including invasive isolates from blood (n = 7), urine (n = 4), ear swabs (n = 2), and pharyngeal swabs (n = 7)] were evaluated. Then, a subset of 89 of erythromycin- and/or clindamycin-resistant strains was assessed in detail, including 82 isolates derived from pregnant women and seven isolates obtained from newborns [blood (n = 4), urine (n = 2), and pharyngeal swabs (n = 1)]. The strains were isolated in the Laboratory of Bacteriology of Obstetrics and Gynecology Clinical Hospital at Poznan University of Medical Sciences (PUMS), from January 2016 to December 2020. The reference strains stored at the Chair and Department of Genetics and Pharmaceutical Microbiology at PUMS were also applied in the assessments.

Specimen collection was performed in the Laboratory of Bacteriology at Gynecological and Obstetric Clinical Hospital PUMS in accordance with recommendations of Centers for Disease Control and Prevention^48^ and the Polish Gynecological Society^49^. Briefly, after incubating the vaginal-rectal swabs in Todd-Hewitt broth with colistin (10 µg/ml) and nalidixic acid (15 µg/ml) (OXOID Deutschland Gmbh, Germany) aerobically at 37 °C for 18–24 h, 10 µl of broth was subcultured on Columbia agar with 5% sheep blood (OXOID Deutschland Gmbh, Germany). Then, selected β- or γ-hemolytic colonies were identified to the species level using a latex agglutination test (OXOID) in accordance with the manufacturer's instructions and based on PCR techniques previously described by Kong et al.^50^. Only one isolate per patient was included in the analysis. Then, the strains were stored for further tests at temperature of − 80 ± 10 °C, in microbanks (Pro-Lab Diagnostics).

### GBS strains DNA extraction

DNA extraction from the isolated GBS strains was carried out using the *in house* developed thermal extraction method^7^. The DNA sample concentrations were measured using the NanoDrop spectrometer (Thermo Scientific) and the DNA samples were stored for further analyses at − 20 ± 2 °C.

### Evaluation of antibiotics susceptibility and mechanisms of resistance to macrolide, lincosamide and streptogramin B

Assessment of antibiotics susceptibility of GBS strains was carried out using the disc diffusion method in accordance with the EUCAST recommendations, applying penicillin (1 IU), erythromycin (15 μg), clindamycin (2 μg), levofloxacin (5 μg), tigecycline (15 μg), tetracycline (30 μg), and levofloxacin (5 μg)^29^. Interpretation of the results of susceptibility testing was performed in accordance with the EUCAST recommendations^29^. Isolates which were phenotypically resistant or intermediate resistant to studied antibiotics were reported as resistant strains. Only strains resistant to erythromycin and/or clindamycin were further investigated.

Resistance to macrolides, lincosamides and streptogramin B was evaluated using the double-disc method in accordance with the EUCAST guidelines^29^. Phenotypes were assigned as follows: (I) inducible mechanism of resistance to macrolide, lincosamide, and streptogramin B (iMLS_B_), when the zone of growth inhibition around the disc with erythromycin indicating the intermediate sensitivity or resistance and flattening of the zone next to the clindamycin halo on the side of erythromycin (so-called D zone) were visible; (II) constitutive mechanism of resistance to macrolide, lincosamide, and streptogramin B (cMLS_B_), when the growth inhibition zone around the erythromycin disc pointing to resistance and the zone of inhibition around the clindamycin disc indicating resistance or intermediate sensitivity were observable; (III) M phenotype (M), when the zone of growth inhibition around the disc with erythromycin indicating the intermediate sensitivity or resistance, and the zone indicating sensitivity around the disc with clindamycin in the absence of flattening zone of growth inhibition were apparent; (IV) L phenotype (L), when the zone of growth inhibition around the disc with clindamycin indicating the intermediate sensitivity or resistance, and the zone indicating sensitivity around the disc with erythromycin were visible.

### Identification of genes related to antibiotic resistance

The presence of the genes associated with antibiotic resistance, erythromycin and/or clindamycin (*ermA*, *ermB*, *mefA*, *lnuB*, and *lsaE*) and tetracycline (*tetM*), was assessed using a duplex or multiplex PCR technique, described in Supplementary Table S1.

### Serotyping and identification of virulence-related genes in macrolide- and/or lincosamide-resistant GBS strains

The use of primers for serotypes Ia, Ib, and II–VII allowed assigning all isolates to single serotypes. Serotypes Ia, Ib, III, and V were identified simultaneously in PCR multiplex reactions, whereas serotypes II, IV, VI, and VII were determined in separate PCR reactions. Supplementary Table S1 presents primers and PCR conditions applied in the serotyping of GBS strains and detection of the *fbsA, fbsB, fbsC* (protein FbsA, FbsB, FbsC), *lmb* (protein Lmb)*, scpB* (C5a peptidase)*, bca,* and *rib* (components of protein C), *alp2/3* (protein Alp 2/3) and *epsilon* (protein Epsilon) virulence-related genes. Identification of hypervirulent ST-17 lineage was performed using a PCR assay based on the detection of the *hvgA* gene^19^. To confirm the specificity of the obtained amplicons, the PCR products were purified using a ExoSAP-IT®for PCR product Clean-Up (Affymetrix) and Sanger sequenced (Genomed, Warsaw, Poland).

### Statistical analysis

Statistical analysis was performed using χ^2^ and Fisher exact tests. *P* values less than 0.05 were considered significant.

### Supplementary Information

Supplementary Table 1.

## Data Availability

The authors confirm that all the data supporting the findings of this study are available within the Article and its Supplementary materials S1.
