# Supplementary material for: Macrolide and lincosamide resistance of Streptococcus agalactiae in pregnant women in Poland
Source: Sci Rep. 2024 Feb 16;14:3877. doi: 10.1038/s41598-024-54521-y (PMC10873391; doi:10.1038/s41598-024-54521-y)
Supplement: Supplementary file 1 — Supplementary Table 1. [file 41598_2024_54521_MOESM1_ESM.docx]

Supplementary Table 1. Primer sequences, product size and annealing temperatures for serotyping, detection of resistance and virulence-associated genes in *S. agalactiae* strains

|  |  | Primer sequence (5’→3’) | Amplicon  (bp) | Annealing temp. (ºC) | Reference |
| --- | --- | --- | --- | --- | --- |
| **Identification** | | | | | |
| GBS cAMP | F  R | TTTTCACCAGCTGTATTAGAAGTA  GTTCCCTGAACATTATCTTTGAT | 155 | 48.0 | (Kong et. al 2005) |
| **Serotyping** |  |  |  |  |  |
| Serotyp Ia | F  R | GGTCAGACTGGATTAATGGTATGC  GTAGAAATAGCCTATATACGTTGAATGC | 521 | 57.0 | (Poyart et. al. 2007) |
| Serotyp Ib | F  R | TAAACGAGAATGGAATATCACAAACC  GAATTAACTTCAATCCCTAAACAATATCG | 770 | 57.0 | (Poyart et. al, 2007) |
| Serotyp II | F  R | GAGAAGCCCAAGGATGGATT  TCCACTATGCTTTTGTAAAATTGG | 188 | 49.2 | DGFM |
| Serotyp III | F  R | CCACATATGAGAATAAGACTTGC  CCAACATACAGACCAATAGTATTC | 187 | 57.0 | (Kong et. al 2005) |
| Serotyp IV | F  R | TTTGCTTGTGAGCTACCTGGA  TCTCCTCCCCAATTTCGTC | 314 | 52.0 | DGFM |
| Serotyp V | F  R | AACAGAGGCCAATCAGTTGC  CTTCCAACGCCACCTCTAAA | 247 | 57.0 | DGFM |
| Serotyp VI | F  R | GGACTTGAGATGGCAGAAGGT  CTCGTACAAACTTCGCTTCCAC | 523 | 54.0 | DGFM |
| Serotyp VII | F  R | GGAGAGAACAATGTCCAGATTACG  AGCTGGTCGTGATTTCTACACA | 369 | 54.0 | DGFM |
| **Virulence genes** | |  |  |  |  |
| *fbsA* | F  R | TCTCAAACCGCAGCGACT  GAAAACAAGAGCCAAGTAGGTCA | 285 | 60.0 | DGFM |
| *fbsB* | F  R | GCTCCGGTTCAATCAGTTGT  GTGGTCATTTCCGCAGTTGT | 137 | 60.0 | DGFM |
| *fbsC* | F  R | CCTGTGAACGCTAAAGCTGAG  CTGTCCTGCCTGAAACAGGT | 397 | 60.0 | DGFM |
| *hvgA* | F  R | ATACAAATTCTGCTGACTACCG  TTAAATCCTTCCTGACCATTCC | 210 | 55.0 | (Lamy et. al 2006) |
| *rib* | F  R | CATACTGAGCTTTTAAATCAGGTGA  TGATACTTCACAGACGAAACAACG | 294 | 55.0 | DGFM |
| *bca* | F  R | TACATGTGGTAGTCCATCTTCACC  TGATACTTCACAGACGAAACAACG | 398 | 55.0 | DGFM |
| *alp2/3* | F  R | TGATACTTCACAGACGAAACAACG  CACTCGGATTACTATAATATTTAGCAC | 334 | 58.0 | (Creti et. al 2004) |
| *epsilon* | F  R | TGATACTTCACAGACGAAACAACG  CCAGATACATTTTTTACTAAAGCGG | 200 | 58.0 | (Creti et. al 2004) |
| *lmb* | F  R | AGTCAGCAAACCCCAAACAG  CTTTCAATTGGCGAGGAGAG | 651 | 58.0 | DGFM |
| *scpB* | F  R | CCTTCCAGACGAAACCAAAA  GCCCTTGTCCTGATTGTCAT | 457 | 58.0 | DGFM |
| *lnuB* | F  R | AACTGGTTGTTTGACGTAGCTC  AGCATAGCCTTCGTATCAGGA | 176 | 58.0 | DGFM |
| *lsaE* | F  R | TACAGCGAGTTGTTTCCTGCT  GTTTTGTTCTCCCACCAAGAAG | 219 | 60.0 | DGFM |
| **Resistance genes** | |  |  |  |  |
| *ermA* | F  R | ATGAGTCAACGGGTGAATGCT  GGTGAAAATATGCTCGTGGCA | 343 | 52.0 | DGFM |
| *ermB* | F  R | GGGCATTTAACGACGAAACTG  ACTTTGGCGTGTTTCATTGC | 539 | 52.0 | DGFM |
| *mefA* | F  R | GCCATTGGTGTGCTAGTGGA  ACCCAATACGTCGATGGCAA | 345 | 52.0 | DGFM |
| *tetM* | F  R | AGAACTAAAAGAGCCTACAG  GTAGTAACATGGTACCCTTT | 609 | 58.0 | DGFM |

DGFM - primers from a set of primers designed at the Department of Genetics and Pharmaceutical Microbiology

Kong, F., Ma, L. & Gilbert, G.L. Simultaneous detection and serotype identification of Streptococcus agalactiae using multiplex PCR and reverse line blot hybridization. *J. Med. Microbiol.* **54**, 1133-1138 (2005)

Poyart, C*. et. al.* Multiplex PCR assay for rapid and accurate capsular typing of group B streptococci. *J. Clin. Microbiol.* **45**, 1985-1988 (2007)

Creti, R., Fabretti, F., Orefici, G., Von Hunolstein, C. Multiplex PCR assay for direct identification of group B streptococcal alpha-like,protein genes. *J. Clin. Microbiol*. **42**, 1326-9 (2004)

Lamy, M.C., Dramsi, S., Billoët, A., Réglier-Poupet, H., Tazi, A., Raymond, J., Guérin, F., Couvé, E., Kunst, F., Glaser, P., Trieu-Cuot, P., Poyart, C. Rapid detection of the "highly virulent" group B Streptococcus ST-17 clone. *Microbes Infect.* **8**, 1714-22 (2006)
